# Development of an Ultrasonication-Assisted Extraction Based HPLC With a Fluorescence Method for Sensitive Determination of Aflatoxins in Highly Acidic *Hibiscus sabdariffa*

**DOI:** 10.3389/fphar.2018.00284

**Published:** 2018-04-06

**Authors:** Xiaofei Liu, Guangyao Ying, Chaonan Sun, Meihua Yang, Lei Zhang, Shanshan Zhang, Xiaoyan Xing, Qian Li, Weijun Kong

**Affiliations:** ^1^Key Laboratory of Bioactive Substances and Resources Utilization of Chinese Herbal Medicine, Ministry of Education, Institute of Medicinal Plant Development, Chinese Academy of Medical Sciences and Peking Union Medical College, Beijing, China; ^2^College of Pharmacy, Jinzhou Medical University, Jinzhou, China

**Keywords:** *Hibiscus sabdariffa*, high acidity, aflatoxins, ultrasonication-assisted extraction, IAC improvement, HPLC-FLD

## Abstract

The high acidity and complex components of *Hibiscus sabdariffa* have provided major challenges for sensitive determination of trace aflatoxins. In this study, sample pretreatment of *H. sabdariffa* was systematically developed for sensitive high performance liquid chromatography-fluorescence detection (HPLC-FLD) after ultrasonication-assisted extraction, immunoaffinity column (IAC) clean-up and on-line post-column photochemical derivatization (PCD). Aflatoxins B_1_, B_2_, G_1_, G_2_ were extracted from samples by using methanol/water (70:30, *v/v*) with the addition of NaCl. The solutions were diluted 1:8 with 0.1 M phosphate buffer (pH 8.0) to negate the issues of high acidity and matrix interferences. The established method was validated with satisfactory linearity (*R* > 0.999), sensitivity (limits of detection (LODs) and limits of quantitation (LOQs) of 0.15–0.65 and 0.53–2.18 μg/kg, respectively), precision (RSD <11%), stability (RSD of 0.2–3.6%), and accuracy (recovery rates of 86.0–102.3%), which all met the stipulated analytical requirements. Analysis of 28 *H. sabdariffa* samples indicated that one sample incubated with *Aspergillus flavus* was positive with aflatoxin B_1_ (AFB_1_) at 3.11 μg/kg. The strategy developed in this study also has the potential to reliably extract and sensitively detect more mycotoxins in other complex acidic matrices, such as traditional Chinese medicines, foodstuffs, etc.

## Introduction

Aflatoxins (AFs) are highly toxic secondary metabolites mainly produced by the toxigenic fungi *Aspergillus flavus* and *Aspergillus parasiticus* that display severe mutagenic, carcinogenic and teratogenic effects (Peraica, 1999; The Joint FAO/WHO Expert Committee on Food Additives, 2012). Aflatoxins are regarded as Group I carcinogens to humans by the World Health Organization [WHO] and International Agency for Research on Cancer [IARC] (1993), and they tend to contaminate a large number of foods and herbal medicines, posing serious threats to the safety of both animals and humans. Because of the detrimental effects of AFs, many countries and organizations have set maximum levels (MLs) for aflatoxin B_1_ (AFB_1_) as well as AFs (sum of aflatoxin B_1_, B_2_, G_1_, G_2_) in a wide range of foodstuffs, herbal medicines and other matrices. The tolerance levels of AFB_1_ set by the European Commission are 5–20 μg/kg in various matrices (European Commission, 2010), while MLs of AFB_1_ and AFs posed by Chinese pharmacopeia (Chinese Pharmacopoeia Commission, 2015) are 5 and 10 μg/kg, respectively.

Because of their acute virulence and wide occurrence combined with ultra-low levels in various matrices, current analytical methods for analysis of AFs mainly include thin layer chromatography (TLC) (Stroka et al., 2009), enzyme-linked immunosorbent assay (ELISA) (Li et al., 2009), and liquid chromatography coupled to tandem mass spectrometry (LC-MS/MS) (Xie et al., 2015; Pesek et al., 2017). However, these methods have several drawbacks including inaccurate quantitation, false-positive results and expensive equipment (Iqbal et al., 2014). Considering the fluorescence quenching properties of AFB_1_ and AFG_1_ in water, the aforementioned drawbacks can be overcome by utilizing high-performance liquid chromatography with fluorescence detection after post-column photochemical derivatization (HPLC-PCD-FLD). This alternative method provided rapid and sensitive detection of AFs in many different types of matrices (Wen et al., 2013; Kong et al., 2014; Kara et al., 2015; Golge et al., 2016; Campos et al., 2017).

Matrix interference is a major challenge for accurate analysis of AFs in traditional Chinese medicines (TCMs) (Bothast and Hesseltine, 1975; Gosetti et al., 2010). Therefore, an appropriate pretreatment step is necessary to purify the sample and enrich for AFs. Liquid-liquid extraction (LLE) (Afzali et al., 2012; Campone et al., 2011; Andrade et al., 2013), matrix solid-phase dispersion (MSPD) (Alessandra et al., 2016), solid-phase extraction (SPE) (Blesa et al., 2003; Alcaide-Molina et al., 2009; Luca et al., 2015; Zhou et al., 2017), quick easy cheap effective rugged and safe (QuEChERS) pretreatment (Desmarchelier et al., 2014) and immunoaffinity column (IAC) methods (Ma et al., 2013; Rhemrev et al., 2015; Wilcox et al., 2015; Xie et al., 2015) have been carried out for multi-matrix cleanup prior to analysis of AFs to eliminate matrix interference. Due to the high selectivity and efficiency, the introduction of IAC can overcome the shortcomings of non-specificity, solvent consumption, and poor efficiency of other cleanup techniques, which has been recommended by the Association of Official Analytical Chemists (AOAC) (Rhemrev et al., 2015). The IAC cleanup technique is primarily based on antibodies that are highly specific to their targets (such as AFs) for sensitive recognition and detection. However, the physical and chemical properties of TCMs, such as their complex makeup varying pH value influence both the stability of antibodies and their ability to recognize their targets, causing low IAC cleanup efficiency and recovery.

The dried calyx and bract of *Hibiscus sabdariffa* L. (family Malvaceae) (Alarcon-Aguilar et al., 2007) are the common ingredients of herbal drinks, hot and cool beverages, and flavoring agents in the food industry. They are also frequently used drugs in clinical trials, where they exhibit anti-bacterial, antifungal, anti-parasitic, anti-pyretic and anti-inflammatory properties (Sindi et al., 2014). *H. sabdariffa* also has high nutritional values due to its large quantities of protein, carbohydrates, fiber, vitamin C and minerals (Mahadevan et al., 2009; Da-Costa-Rocha et al., 2014; Sindi et al., 2014). Drinking of *H. sabdariffa* tea daily has a positive impact on blood pressure in type II diabetic patients (Mozaffari-Khosravi et al., 2009) and reduces hypercholesterolaemia (Aziz et al., 2013). However, the safety and quality of TCMs are threatened by mold and various fungi that can thrive under the high temperatures and humidity condition during the growth, processing, storage and transportation progresses, which can lead to aflatoxin contamination (Wang et al., 2012; Akhund et al., 2017). And the occurrence of aflatoxins contamination in many matrices including herbal medicines of leaves and flowers has been reported (Han et al., 2010, 2012; Tan et al., 2012; Zheng et al., 2014). It is also investigated that *H. sabdariffa* samples from Uyo and Akpan markets were contaminated by AFB_1_ (Adebayo-tayo and Samuel, 2009). Therefore, it is of great urgency to develop reliable methods for sensitive detection of aflatoxins in *H. sabdariffa*. But, the high acidity and complex matrix interferences have been obstacles for *H. sabdariffa* sample preparation and clean-up for detection of trace aflatoxins.

In this study, we describe ideal pretreatment conditions that account for the high acidity and complex compon ents of *H. sabdariffa* samples. We systematically optimized the sample pretreatment procedure including extraction technique, the dilution ratio of sample solutions and the buffer dilution solution. This is the first report on detection of aflatoxins by HPLC-PCD-FLD in acidic sample like *H. sabdariffa*. This strategy provides essential guidance for monitoring toxic contaminates in *H. sabdariffa* and can be applied to other highly acidic TCMs and foodstuffs, and feeds, etc.

## Materials and Methods

### Protection and Safety Statement

Due to the extremely strong toxicity of aflatoxins, necessary cautions and essential protections are needed throughout the whole experiments according to strict guidelines. Anti-poison respirators, isolation gowns and goggles were used. After being contaminated with aflatoxins, the containers and consumables were dipped in 10% sodium hypochlorite in a specified holder for at least 24 h. The lab garbage was disposed in an appropriate way.

### Reagents and Chemicals

The IAC-ToxinFast^®^-Aflatoxins Test IACs were supplied by Huaan Magnech Bio-Tech Co., Ltd. (Beijing, China). The TGL 16M centrifuge was bought from Hunan Kaida Scientific Instrument Co., Ltd. (Changsha, China) and PB-10 pH meter from Sartorius Intec (Hamburg, Germany). HPLC grade methanol and acetonitrile were purchased from Fisher Scientific (Fair Lawn, NJ, United States). Other reagents and chemicals of analytical grade were obtained from Beijing Chemical Works (Beijing, China). Deionized water was purified by the Milli-Q Plus ultra-pure water system (Millipore, Billerica, MA, United States).

Phosphate buffer saline (PBS) was prepared by dissolving 0.2 g KCl, 0.2 g KH_2_PO_4_, 8.0 g NaCl, and 1.2 g Na_2_HPO_4_ in 1000 mL of pure water at pH 7.4. 0.1 M NaH_2_PO_4_ solution was set as Solution A, which was obtained by dissolving 1.56 g NaH_2_PO_4_⋅2H_2_O in 100 mL of pure water, while Solution B was obtained by dispersing 35.8 g Na_2_HPO_4_⋅12H_2_O in 1000 mL of pure water. 0.1 M Phosphate buffer (PB), encompassing 60 mL of Solution A and 940 mL of Solution B, was homogeneously mixed and afterward the pH value was adjusted to 8.0 with 0.1 M HCl. 0.5%, 1%, and 2% PBST (PBS with addition of Tween-20) solutions were got by adding 5, 10 and 20 mL of Tween-20 into 1000 mL PBS (pH 7.4), respectively.

### Instrumentation and HPLC Conditions

All analyses were performed on a Shimadzu LC-20AT HPLC system (Shimadzu, Kyoto, Japan) consisting of two LC-20 AT pumps, an SIL-20A autosampler, a CTO-20A column oven, a CMB-20A controller, the post-column PCD reactor and an RF-20AXL fluorescence detector. The PCD reactor with a mercury lamp (λ = 254 nm) and a knitted reactor coil of 0.74 mL (15 m × 0.25 mm) was bought from AURA Industries (New York, NY, United States). The eluate was monitored by using a fluorescence detector with an excitation wavelength of 360 nm and an emission wavelength of 450 nm. The chromatographic separation of tested aflatoxins was conducted on a CAPCELL PAK C18 column (4.6 mm ID × 150 mm, 5 μm) at constant 30°C. HPLC separation of four AFs was carried out by using methanol and acetonitrile (40: 18, *v/v*) with the water phase (pure water) as the mobile phase at isocratic elution. Injection volume was 20 μL and the flow rate was set at 1.2 μL/min. The total run time was 30 min.

### Working Standard Solution

Aflatoxins standard including 2 μg AFB_1_, 2 μg AFG_1_, 0.5 μg AFB_2_, 0.5 μg AFG_2_ in 1 mL of acetonitrile were bought from Pribolab (Singapore). Working standard solution was prepared by diluting the standards with methanol to get 200 ng/mL of AFB_1_, AFG_1_ and 25 ng/mL of AFB_2_, AFG_2_. All the standard solutions were stored at -20°C in the dark before analysis.

### Sample Collection

Twenty-six batches of *H. sabdariffa* samples including 6 batches of ground powder (S1–S6), 11 batches of crude materials (S7–S17) and 9 kinds of scented teas (S18–S26) were randomly collected from Guangxi, Sichuan, Anhui, Guangdong and Yunnan provinces, Beijing city of China and Thailand. 500 g sample was used for the preparation of sample solution. A positive control (S28) was obtained by inoculating the sample from Beijing city with *A. flavus* spores while another fraction of this sample (S27) was kept at the same incubation conditions without *A. flavus*. All crude samples and scented teas were identified by Prof. Meihua Yang (Institute of Medicinal Plant Development, Chinese Academy of Medical Sciences, Peking Union Medical College). All the samples were thoroughly triturated to obtain consistent particle size and homogenized samples. The sub-sample and scented teas were powdered and sieved through a 24-mesh sieve. All samples were classified and labeled clearly in lock bags while stored in a cool dry place before use.

### Sample Preparation

#### Fungal Spore Suspension Preparation

The *A. flavus* spore suspensions were prepared according to a previously reported method (Christensen et al., 2012) with some modifications. After 1 week of incubation, 10.0 mL of sterile water was spiked onto the Czapek Dox Agar (CDA) medium (Aobox Biotechnology Co., Ltd., Beijing, China) with *A. flavus*. After standing for 1 min, the yellow-green spore was scraped down with a sterile inoculating loop. After mixed with sterile glass beads in a 15-mL aseptic centrifuge tube, the spore suspension was diluted with sterile water to 1 × 10^5^ spores/mL as determined by using a haemocytometer slide (0.1 mm depth, 1/400 mm^2^) and XDS-1B optical microscope (Chongqing COIC Instrument Co., Ltd., Chongqing, China).

#### Preparation of Artificial Moldy Samples

Approximately 20.0 g of the *H. sabdariffa* sample purchased from Beijing city was sterilized under an ultraviolet lamp for 1 h. Then, the sterilized sample was inoculated with *A. flavus* spores by *trans*-inoculation to produce a contaminated *H. sabdariffa* control. Inoculation was performed as follows: 50 mL of *A. flavus* spore suspension (1 × 10^5^ spores/mL) was added onto the surface of the sample, which was covered and incubated for 30 min at constant humidity and temperature (90% relative humidity, 28°C). Afterwards, spore suspension was removed and the sample was incubated under the same conditions for 20 days. A sample that was not inoculated with the *A. flavus* was also incubated for 20 days (90% relative humidity, 28°C). The moldy *H. sabdariffa* sample was powdered and stored at -20°C in the freezer for further utilization.

#### Extraction Procedure

The described method in Chinese Pharmacopeia (Chinese Pharmacopoeia Commission, 2015) after some modifications was used. *H. sabdariffa* (5.00 g ± 0.01 g) was mixed in a 50-mL centrifuge tube with 1.00 g NaCl and 25 mL of methanol/water (70:30, *v/v*) solution. The mixture was homogenized by swirling for 2 min, and then was extracted by ultrasonication in an ultrasonic bath at 500 W for 20 min. The slurry was separated at 10,000 rpm for 5 min at 20°C. Immediately after 5 mL of the upper phase was transferred into a new centrifuge tube, 40 mL of 0.1 M PB (pH 8.0) was added to dilute the sample 1:8. The diluent was filtered through 0.45-μm glass fiber filter paper for the next cleanup step.

### Immunoaffinity Column Cleanup

The resulting 40 mL of filtrate was passed through an immunoaffinity column (IAC-ToxinFast^®^-Aflatoxins Test) at a flow rate of approximately 3 mL/min at room temperature for at least 30 min. When the filtrate nearly passed through the column, 20 mL of ultra-pure water was added to wash the column at a flow rate of 3 mL/min. Finally, all the residual liquid was excluded with clean air. Next, 1.5 mL of methanol was added onto the IAC for elution of the aflatoxins. The final volume of the eluate was brought to 2 mL with methanol. After being mixed uniformly, the final solution was filtered through a 0.22-μm glass fiber filter paper.

### Analytical Measurement

Sensitive quantitation of four AFs in *H. sabdariffa* samples in the form of ground powder, crude herbs and scented teas was carried out by measuring the peak area responses in the HPLC-FLD chromatograms at eachg aflatoxin retention time and comparing them with the linear equation of a calibration graph. The peak area (*y-*axis) of each aflatoxin was plotted against the concentration (ng/mL, *x-axis*) and expressed as a linear equation *y* = *ax*+ *b*. By determining the values of slope (*a*) and *y-*intercept (*b*), the equations were established and the content of each aflatoxin in the tested sample was calculated.

### Method Validation

For accurate determination of four target aflatoxins in *H. sabdariffa* samples with high acidity, the established HPLC-PCD-FLD method was validated according to EU Regulation No. 401/2006 (The Commission of the European Communities, 2006) in terms of linearity, limit of detection (LOD) and limit of quantitation (LOQ), accuracy, precision, stability, selectivity and robustness.

### Statistical Analysis

Each experiment was performed in triplicate and all the data were represented as mean ± standard error (SE) and analyzed by ANOVA using SPSS software (version 19.0). Means were separated by Tukey’s multi-range tests when ANOVA results were significant (*P* < 0.05).

## Results and Discussion

This work aimed to develop a simple, rapid and sensitive method for reliable determination of four aflatoxins in the acidic sample, *H. sabdariffa* by HPLC-FLD after ultrasonication-assisted extraction, IAC cleanup, and online post-column PCD. Some key conditions and parameters for sample preparation and IAC cleanup were optimized to amend the acidity by decreasing pH and eliminate impurities in the samples.

### Sample Pretreatment Optimization

Results of preliminary experiments using the recommended method (Chinese Pharmacopoeia Commission, 2015) for detection of AFs in *H. sabdariffa* showed that the four AFs (AFB_1_, AFB_2_, AFG_1_, AFG_2_) were not adequately separated and recovered. Unfavorable detection was due to complex matrix components and the high acidity of this TCM. To overcome these issues, we modified and improved sample pretreatment and cleanup for sensitive HPLC-FLD analysis. First of all, necessary work was reasonably performed on different dilution ratios of pure water. The sample extract was diluted with pure water at dilution ratios of 1:4 and 1:8 (*v/v*). These dilutions were inadequate to recoveries of AFs at the required levels of 70–110%. Therefore, other highly effective dilution reagents were taken into consideration.

### Selection of the Buffer Dilution Solution

The optimal pH value for IACs is in a range of 6.0–8.0. The mean pH value of the above-mentioned diluent in water at 1:8 ratio was around 2.4, which will weaken the stability of antibodies and their target affinity. Few immune reactants would be out of function under this condition. Diluting the sample just with water to provide buffer capacity and minimize the matrix was not sufficient to overcome the strongly acidic matrix of *H. sabdariffa* (Da-Costa-Rocha et al., 2014).

**FIGURE 1:**
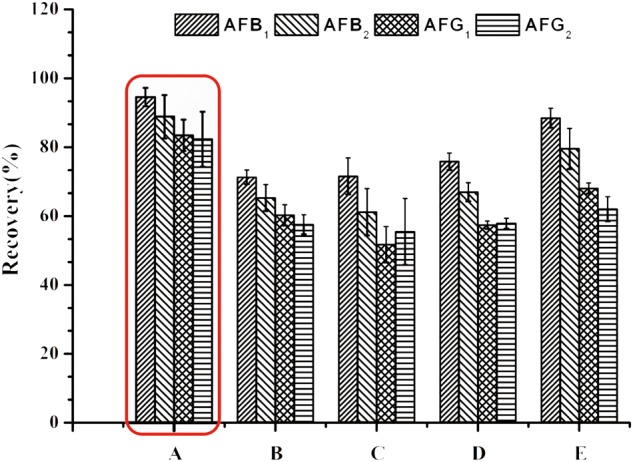
Recoveries of spiked *H. sabdariffa* samples with 5.0 μg/kg of AFB_1_ and AFG_1_, 1.25 μg/kg of AFB_2_ and AFG_2_ in different dilution solutions of **(A)** 0.1 M PB (pH 8.0); **(B)** PBS (pH7.4); **(C)** PBS (pH7.4)-0.5% Tween-20; **(D)** PBS (pH7.4)-1% Tween-20; **(E)** PBS (pH7.4) -2% Tween-20.

To resolve the problem, PBS (Ip and Che, 2006) was introduced to adjust the pH of the test solution before application onto the IACs. Unfortunately, it was found that the recoveries of AFG_1_ and AFG_2_ still did not meet the official requirements. To address this issue, Tween-20 (Zhang et al., 2017) was added into the buffer, and three levels (0.5, 1 and 2%, *v/v*) of Tween-20 were compared to screen the most effective buffer dilution solution. Increasing the volume of Tween-20, the recoveries of AFs went up mildly (**Figure 1**). However, even with 2% Tween-20 in PBS (pH 7.4) the recovery of AFG_2_ did not meet the requirement.

For extracts of *H. sabdariffa* with such high acidity (around pH3.0), 0.1 M PB (pH 8.0), which has a greater buffering capacity, could be used to successfully adjust the pH to the appropriate range (pH > 6.0) for IAC. When 0.1 M PB (pH 8.0) was used for dilution, the recoveries of four aflatoxins in *H. sabdariffa* increased sharply to acceptable levels (**Figure 1**).

By diluting samples with 0.1 M PB (pH 8.0), matrix interferences were minimized and AFs recoveries were within the standard range. Therefore, to ensure that the concentrations of AFs in the final eluates could successfully reach the LODs and to decrease useless hands-on time, a dilution ratio of 1:8 (*v/v*) was finally chosen.

### Optimization of Sample Extraction Technique

**FIGURE 2:**
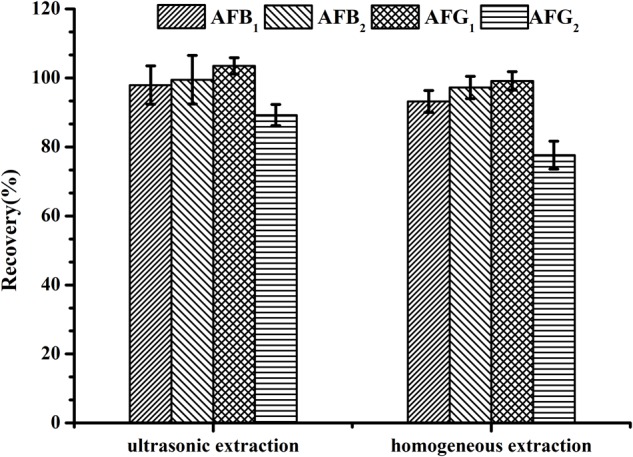
Recoveries of spiked *H. sabdariffa* samples with 5.0 μg/kg of AFB_1_ and AFG_1_, 1.25 μg/kg of AFB_2_ and AFG_2_ using different extraction techniques.

Using a suitable sample extraction technique plays an important role in trace detection for effectively extracting aflatoxins in *H. sabdariffa* without increasing impurities. A homogeneous extraction technique has been recommended (Chinese Pharmacopoeia Commission, 2015). Here, two simple and commonly used techniques including homogeneous extraction for 3 min at 11,000 rpm with a homogenizer and ultrasonication-assisted extraction in an ultrasonic bath at 500 W for 20 min were both tested to compare their efficacy in extracting AFs from spiked *H. sabdariffa* samples with 5.0 μg/kg of AFB_1_ and AFG_1_, and 1.25 μg/kg of AFB_2_ and AFG_2_. Recoveries of the four AFs in spiked *H. sabdariffa* samples were all over 80%, but the values obtained from ultrasonication-assisted extraction were all higher than that from homogeneous extraction (*P* < 0.05) (**Figure 2**). We found that after homogeneous treatment, a large quantity of the sample solution was adhered to the surface of the stirring arm. In addition, with this method, samples were processed sequentially and the machinery had to be washed carefully between testing, resulting in wasted analytes and time. Therefore, ultrasonication-assisted extraction using 70% methanol was chosen for sample preparation. With the application of modifications, the recoveries of four aflatoxins met the trace analysis requirements.

### Method Validation

The method was validated for linearity, LOD and LOQ, accuracy, precision, stability, selectivity and robustness described as follows.

#### Linearity and LOD, LOQ

The standard curve for each aflatoxin was performed by a serial concentration of the standard solutions. Six concentrations of AFB_1_ and AFG_1_ (0.625, 1.25, 2.5, 5.0, 10.0, 20.0 ng/mL) and five concentrations of AFB_2_ and AFG_2_ (0.3125, 0.625, 1.25, 2.50, 5.0 ng/mL) were generally diluted and performed in triplicate for the linearity of each aflatoxin, respectively. Four independent linear relationships of instrument response (area of target peak, *y*) and target concentration (*x*) satisfactorily showed in appropriate detection ranges (**Table 1**) with correlation coefficients (*R*) > 0.999.

**Table 1:** Linearity, LOD and LOQ for tested aflatoxins.

Aflatoxins	Linear equation	*R*	Ranges (ng/mL)	LOD (μg/kg)	LOQ (μg/kg)
AFB_1_	*y* = 28412 *x* + 5917.8	0.9998	0.625–20	0.18	0.60
AFB_2_	*y* = 61720 *x* + 2983.0	0.9998	0.3125–5	0.15	0.53
AFG_1_	*y* = 16589 *x* + 3386.8	0.9998	0.625–20	0.65	2.18
AFG_2_	*y* = 36708 *x* + 1706.2	0.9997	0.3125–5	0.41	1.22

Limits of detection (LODs) and limits of quantitation (LOQs) were carried out by serial dilution of standard solutions, which referred to the concentrations of the standards when the ratios of signal of chromatographic peak to noise (signal-to-noise ratio, S/N) were 3 and 10, respectively. The S/N ratios were calculated by Analyst Software (version 1.6.2). The LODs and LOQs were between 0.15–0.65 μg/kg and 0.53–2.18 μg/kg, respectively (**Table 1**), which were below the MLs defined in EU and Chinese pharmacopeia (2015 edition). So, the method was sensitive enough for HPLC-FLD analysis.

#### Accuracy, Precision and Stability

The accuracy of the established HPLC-FLD method was assessed in recovery at three levels of aflatoxins in normal (aflatoxins-free) samples. The recoveries at three levels (low, medium and high concentrations in **Table 2**) were 86.0–98.9%, 90.7–102.3% and 95.0–101.8%, respectively, which met the official requirements.

**Table 2:** Precision, recoveries and stability for *Hibiscus sabdariffa* spiked with four aflatoxins.

Aflatoxins	Precision (RSD%, *n* = 6)		Recoveries (%) (*n* = 6)			Stability (RSD%)
	Intra-day	Inter-day	High level^a^	Medium level^b^	Low level^c^	
AFB_1_	9.1	5.3	90.1 ± 7.2	97.6 ± 7.9	95.5 ± 3.1	0.2
AFB_2_	7.3	4.5	92.1 ± 5.0	101.0 ± 7.2	99.6 ± 1.5	3.6
AFG_1_	6.3	7.3	98.9 ± 5.3	102.3 ± 5.1	101.8 ± 5.2	0.9
AFG_2_	10.0	4.4	86.0 ± 3.3	90.7 ± 2.1	95.0 ± 4.0	2.3

*^*a*^10 μg/kg of AFB_*1*_, AFG_*1*_, 2.5 μg/kg of AFB_*2*_ and AFG_*2*_. ^*b*^5 μg/kg of AFB_*1*_, AFG_*1*_, 1.25 μg/kg of AFB_*2*_ and AFG_*2*_. ^*c*^2.5 μg/kg of AFB_*1*_, AFG_*1*_, 0.625 μg/kg of AFB_*2*_ and AFG_*2*_.*

The normal sample was spiked with a 5 μg/kg standard solution for evaluating method precision. Precision data (RSD%) was estimated by repeated analysis (*n* = 6) of the fortified sample spiked with AFs at the MLs concentration. Intra- and inter-day precisions analyses were also carried out (**Table 2**). RSD values of 6.3–10.2% (*n* = 6) and 4.4–7.3% (*n* = 6, 6 days) were below the references in the European Commission Regulations, showing good precision of the developed method.

Additionally, the stability of a series of AFs standard solutions (stored at 4°C for 1 week in the dark) was measured every day (7 days in total). Repeated results (RSDs 0.2–3.6%) showed that the standard solutions of four aflatoxins (AFB_1_, AFB_2_, AFG_1_, and AFG_2_) were stable under these circumstances for 1-week storage (**Table 2**).

#### Selectivity and Robustness

**FIGURE 3:**
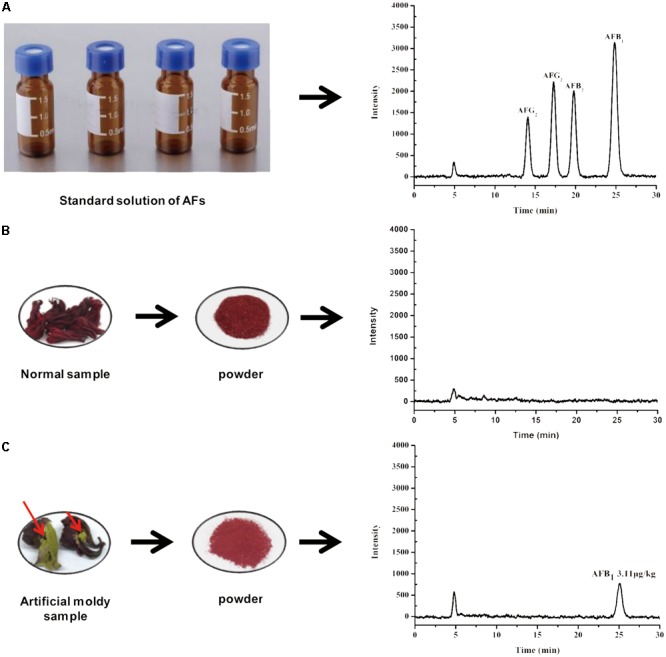
Typical HPLC-FLD chromatograms of **(A)** AFs standard solution, **(B)** normal sample, and **(C)** artificial moldy *H. sabdariffa* sample positive with AFB_1_.

The selectivity of the developed HPLC-FLD method for sample clean-up and enrichment was evaluated by IAC. The immuno-adsorption between AFs and antibodies indicated that the IAC method with post-column PCD was selective. After optimization of the chromatographic conditions mentioned above, the chromatograms exhibited the expected results for the mixed standard solution. AFs in the fortified sample with 5.0 μg/kg of AFB_1_ and AFG_1_, 1.25 μg/kg of AFB_2_ and AFG_2_ were effectively separated in 27 min (**Figure 3**). There were no interference peaks at the retention time when each target aflatoxin was observed.

Robustness, as an index for variations, remained unaffected by small but notable variations during the testing procedure. In this assay, the slight variations of water composition (±0.01%) in mobile phase and in column temperature above or below 0.2°C did not affect the results. Therefore, our newly technique was robust.

### Real Samples Analysis

To confirm the suitability and versatility of our optimized pretreatment conditions including ultrasonication-assisted extraction and IAC cleanup, the validated HPLC-FLD analytical procedure after online PCD was applied for simultaneous determination of four AFs in 28 *H. sabdariffa* samples including 6 batches of ground powders, 11 batches of crude materials and 9 kinds of scented teas purchased from different markets, herbal shops and bazaars in China and Thailand, and two man-made moldy samples. The HPLC-FLD chromatograms showed that the four AFs were well separated with satisfactory responses under the present chromatographic conditions (**Figure 3**). No interference peaks were detected at the retention time of each aflatoxin in the normal (AFs-free) and moldy samples. The occurrence and contents of four AFs in different *H. sabdariffa* samples for this survey were summarized in **Table 3**. One artificial moldy control sample after being inoculated with *A. flavus* spores for 20 days was tested to be positive for AFB_1_ at 3.11 μg/kg, which did not exceed the MLs set by China and other international organizations. Because of its important medicinal values and world-wide consumption, it is important to monitor the occurrence and levels of aflatoxins especially AFB_1_ in *H. sabdariffa* products to ensure quality and safety.

**Table 3:** Occurrence and contents of four AFs in different *H. sabdariffa* samples from different origins.

Sample property	No.	Origin	Aflatoxins (μg/kg)			
			AFB_1_	AFB_2_	AFG_1_	AFG_2_
Grounded powder	S1	Anhui	–^a^	–	–	–
	S2	Anhui	–	–	–	–
	S3	Zhejiang	–	–	–	–
	S4	Fujian	–	–	–	–
	S5	Anhui	–	–	–	–
	S6	Guangdong	–	–	–	–
Crude materials	S7	Zhejiang	–	–	–	–
	S8	Guangdong	–	–	–	–
	S9	Guangxi	–	–	–	–
	S10	Yunnan	–	–	–	–
	S11	Fujian	–	–	–	–
	S12	Sichuan	–	–	–	–
	S13	Yunnan	–	–	–	–
	S14	Thailand	–	–	–	–
	S15	Shandong	–	–	–	–
	S16	Beijing	–	–	–	–
	S17	Guangxi	–	–	–	–
Scented tea	S18	Fujian	–	–	–	–
	S19	Anhui	–	–	–	–
	S20	Zhejiang	–	–	–	–
	S21	Fujian	–	–	–	–
	S22	Yunnan	–	–	–	–
	S23	Anhui	–	–	–	–
	S24	Jiangsu	–	–	–	–
	S25	Shandong	–	–	–	–
	S26	Beijing	–	–	–	–
Artificial moldy sample	S27	Beijing	–	–	–	–
	S28	Beijing	3.11	–	–	–

*^*a*^Not detected.*

## Conclusion

In this study, by systematically optimizing the sample pretreatment conditions including the dilution ratio, type of buffer and the sample extraction technique, a simple, sensitive and rapid HPLC-PCD-FLD method after IAC cleanup has been developed for simultaneous determination of four AFs in different types of *H. sabdariffa* samples. The established HPLC-PCD-FLD method posted sensitive and accurate detection of aflatoxins to overcome the major analytical challenges of high acidity and low concentration of targets in *Hibiscus sabdariffa*. Compared to other pretreatment procedures, the modified extraction and suitable IAC clean-up method can achieve highly effective recognition of trace AFs in highly acidic *H. sabdariffa* samples, leading to satisfactory LODs for the targets.

After systematic optimization, the developed method should be applicable for the sensitive detection of more mycotoxins in other types of TCM matrices of high acidity to ensure their quality and safety. In addition, the elucidation of fungal behavior, together with the influence of mycotoxins contamination on the quality of *H. sabdariffa* is a relevant focus in the future.

## Author Contributions

XL wrote the paper. GY, WK, and XL conceived and designed the experiments. SZ, LZ, and QL performed the sample preparation and chemical pretreatments. GY and CS contributed to preparing artificial moldy samples. XL, GY, and XX gave rise to analyzing the data. MY and WK reviewed the paper and funded the project. WK was responsible for submission.

## Conflict of Interest Statement

The authors declare that the research was conducted in the absence of any commercial or financial relationships that could be construed as a potential conflict of interest.
